# Supplementary material for: DMRforPairs: identifying Differentially Methylated Regions between unique samples using array based methylation profiles
Source: BMC Bioinformatics. 2014 May 15;15:141. doi: 10.1186/1471-2105-15-141 (PMC4046028; doi:10.1186/1471-2105-15-141)
Supplement: Additional file 2 — DMRforPairs output for the comparison of A431-MCF7 and NA17018-NA17105. Please start from the HTML files in each folder. Available via the BMC Bioinformatics website. [file 1471-2105-15-141-S2.zip › 1394847754114233_MOESM2_ESM/A431_MCF7/figures/33975.pdf]

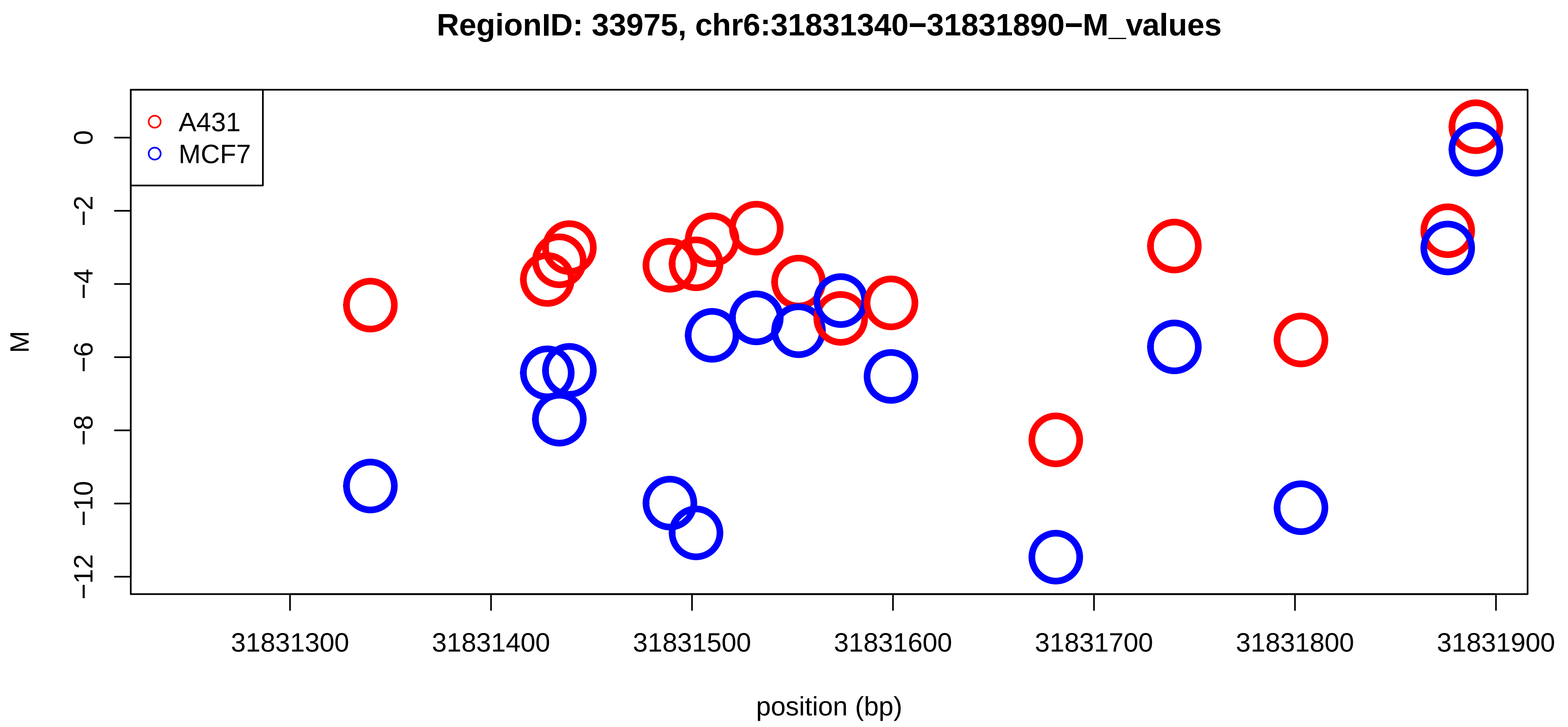
RegionID: 33975, chr6:31831340−31831890−M_values
A431
MCF7
M
0
−2
−4
−6
−8
−10
−12
31831300
31831400
31831500
31831600
31831700
31831800
31831900
position (bp)

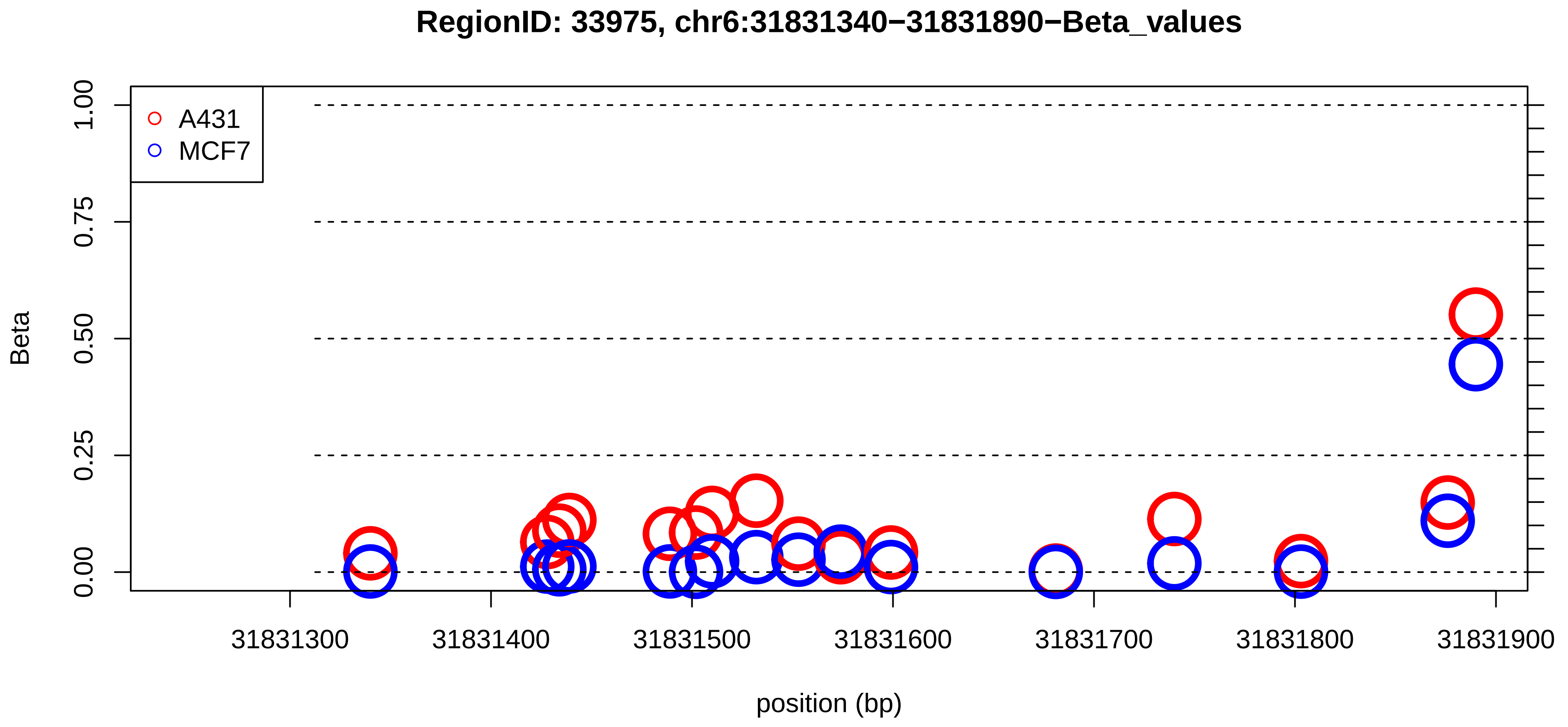
RegionID: 33975, chr6:31831340−31831890−Beta_values
A431
MCF7
Beta
1.00
0.75
0.50
0.25
0.00
31831300
31831400
31831500
31831600
31831700
31831800
31831900
position (bp)

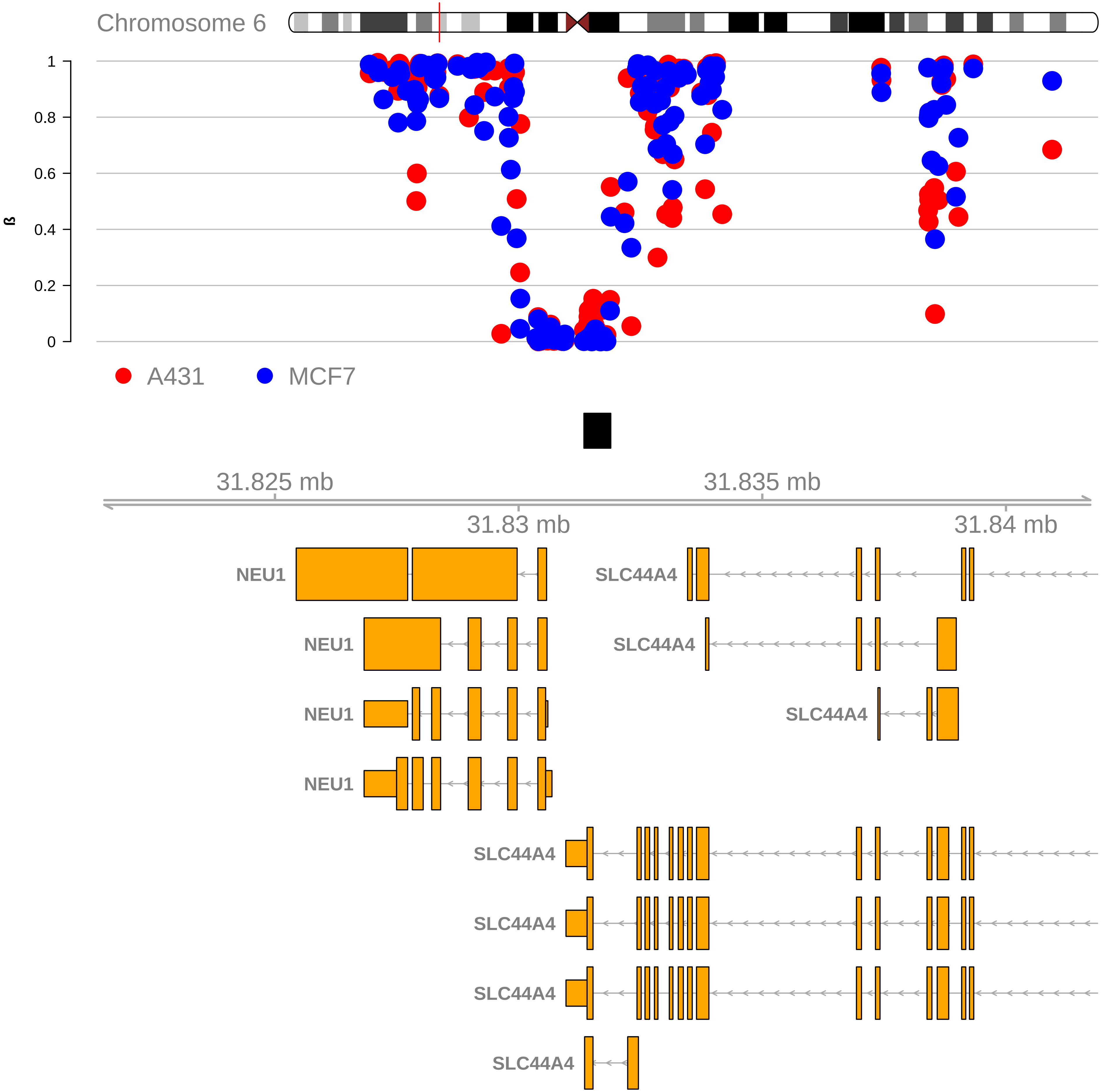

Chromosome 6
ß
1
0.8
0.6
0.4
0.2
0
A431
MCF7
31.825 mb
31.835 mb
31.83 mb
31.84 mb
NEU1
SLC44A4
NEU1
SLC44A4
NEU1
SLC44A4
NEU1
SLC44A4
SLC44A4
SLC44A4
SLC44A4
